# Supplementary material for: The Lyotropic Nature of Halates: An Experimental Study
Source: Molecules. 2022 Dec 3;27(23):8519. doi: 10.3390/molecules27238519 (PMC9739596; doi:10.3390/molecules27238519)
Supplement: Supplementary file 1 [file molecules-27-08519-s001.zip › molecules-2024092-supplementary.pdf]

The Lyotropic Nature of Halates: An Experimental Study

Mert Acar [1], Duccio Tatini [1], Barry W. Ninham [2,3], Federico Rossi [4], Nadia Marchettini [4] and Pierandrea Lo Nostro [1,*]

[1] Department of Chemistry "Ugo Schiff" and CSGI, University of Florence, 50019 Firenze, Italy
[2] Materials Physics (Formerly Department of Applied Mathematics), Research School of Physics, Australian National University, Canberra, ACT 2600, Australia
[3] School of Science, University of New South Wales, Northcott Drive, Campbell, Canberra, ACT 2612, Australia
[4] Department of Earth, Environmental and Physical Sciences, University of Siena, 53100 Siena, Italy
* Correspondence: pierandrea.lonostro@unifi.it; Tel.: +39-055-4573010

Supplementary Information

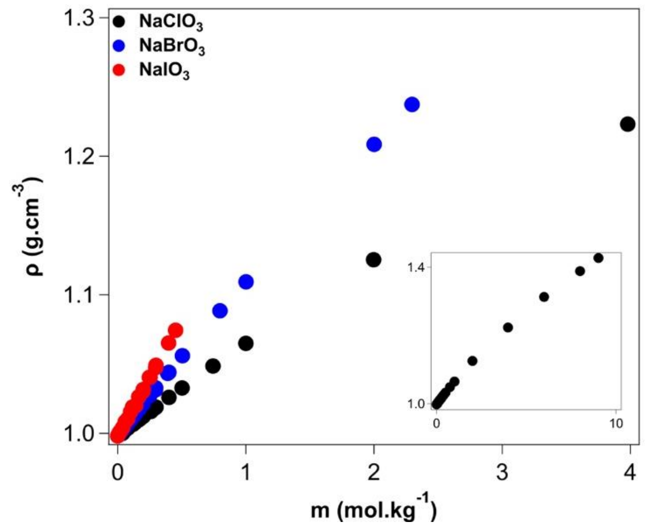

Figure S1. Density ρ as a function of the salt concentration (m, in molal units) for sodium chlorate (black), bromate (blue) and iodate (red) solutions at 20° C. The inset shows the full investigated concentration range for $NaClO_3$.

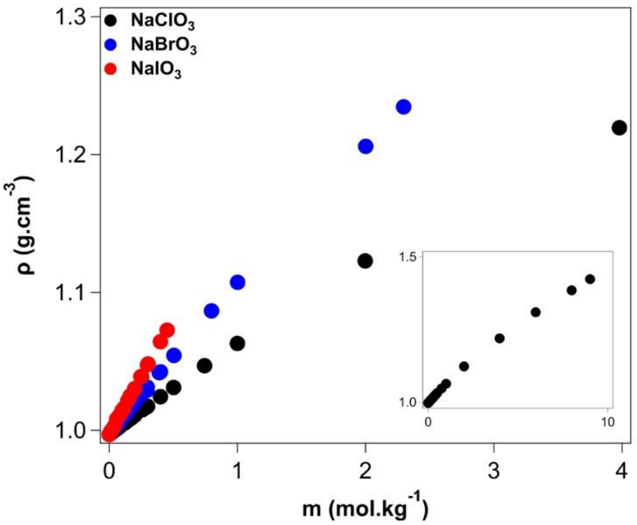

Figure S2. Density ρ as a function of the salt concentration (m, in molal units) for sodium chlorate (black), bromate (blue) and iodate (red) solutions at 25° C. The inset shows the full investigated concentration range for $NaClO_3$.

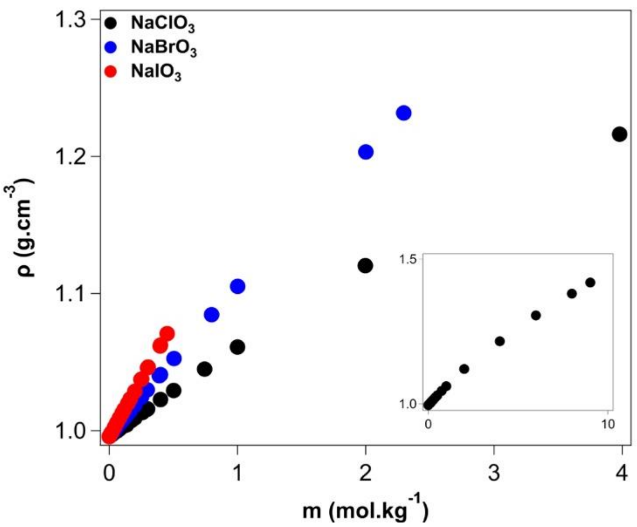

Figure S3. Density ρ as a function of the salt concentration (m, in molal units) for sodium chlorate (black), bromate (blue) and iodate (red) solutions at 30° C. The inset shows the full investigated concentration range for $NaClO_3$.

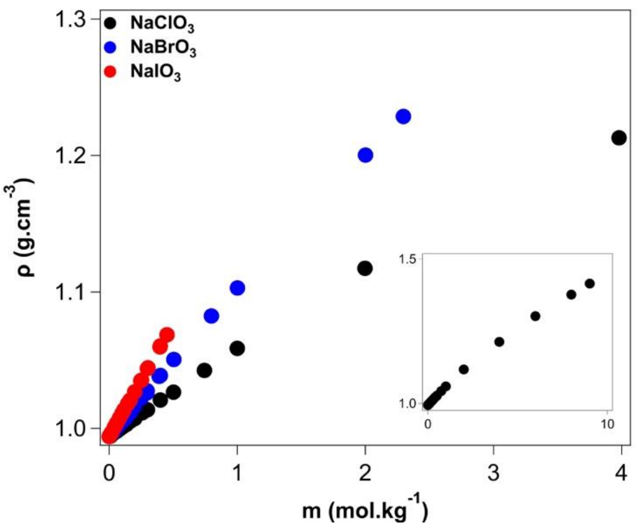

Figure S4. Density ρ as a function of the salt concentration (m, in molal units) for sodium chlorate (black), bromate (blue) and iodate (red) solutions at 35° C. The inset shows the full investigated concentration range for $NaClO_3$.

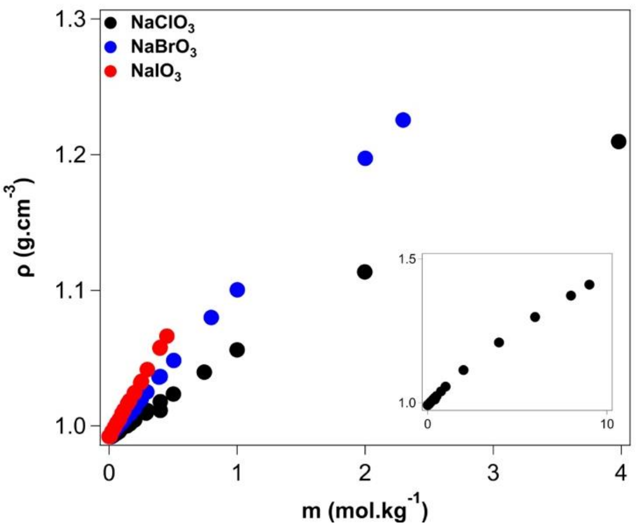

Figure S5. Density ρ as a function of the salt concentration (m, in molal units) for sodium chlorate (black), bromate (blue) and iodate (red) solutions at 40° C. The inset shows the full investigated concentration range for $NaClO_3$.

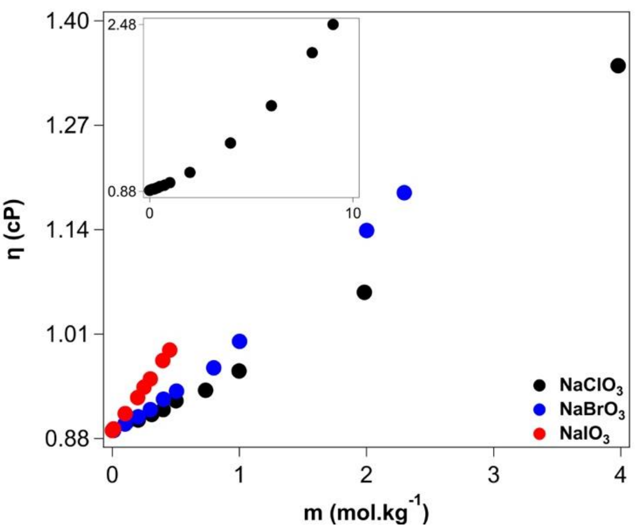

Figure S6. Viscosity η (in cP) as a function of the concentration (m, in molal units) at 25° C of sodium chlorate (black), bromate (blue) and iodate (red) aqueous solutions. The inset shows the full concentration range investigated for $NaClO_3$.

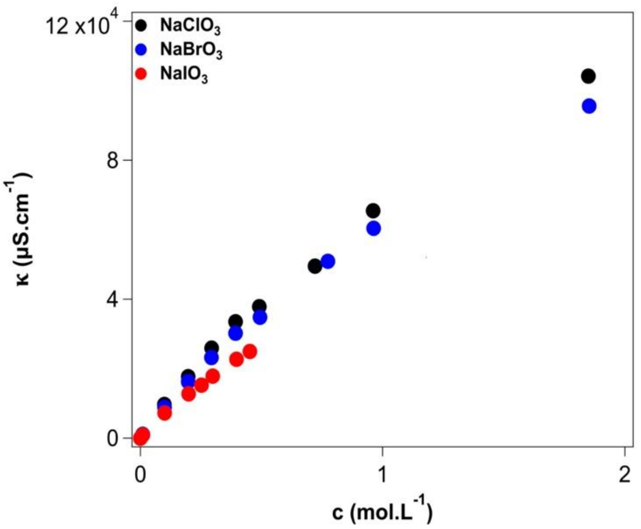

Figure S7. Conductivity κ (in $\mu S \cdot cm^{-1}$) at 25° C of sodium chlorate (black), bromate (blue) and iodate (red) aqueous solutions as a function of concentration (c, in molar units).

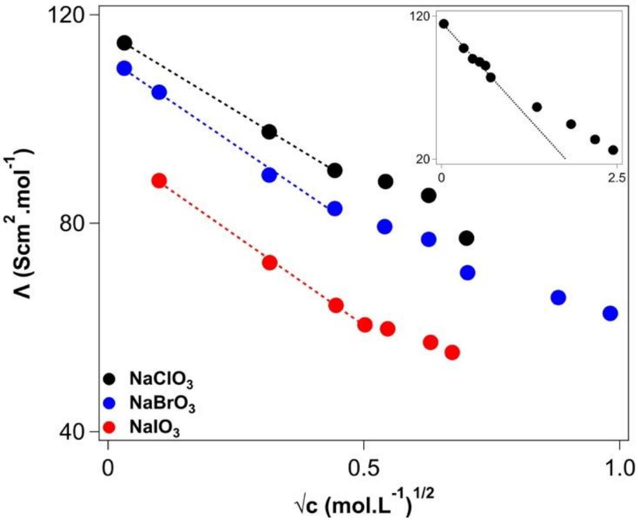

Figure S8. Molar conductivity Λ as a function of the square root of the concentration of sodium chlorate (black), bromate (blue) and iodate (red) solutions. Dotted lines represent the fitting to eq. A10. The experimental absolute error on the molar conductivity values is ± 0.1.

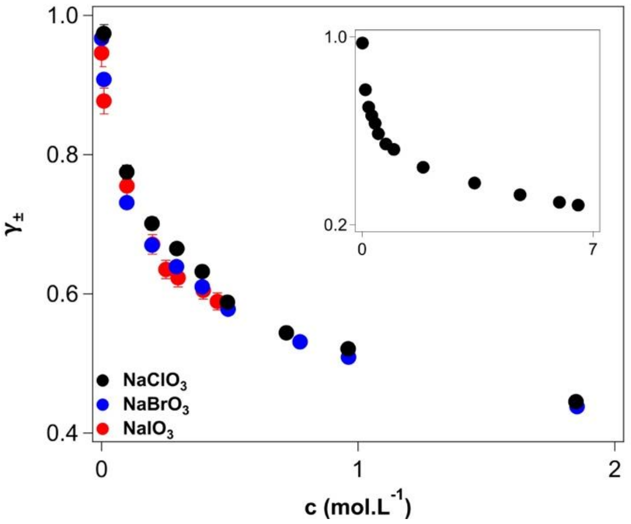

Figure S9. Mean ionic activity coefficients ($\gamma_\pm$) at 25° C of sodium chlorate (black), sodium bromate (blue) and sodium iodate (red) aqueous solutions as a function concentration (c, in molar units) calculated by eq. A9. The error is of ±1.3%, ±0.6% and ±2.1% for sodium chlorate, bromate and iodate, respectively.

Table S1. Density (ρ, in g·cm^{-3}) at 20° C of sodium chlorate, sodium bromate and sodium iodate solutions at different concentrations (m, in molal units). σ is the standard deviation.

$NaClO_3$			$NaBrO_3$			$NaIO_3$		
m	ρ	σ	m	ρ	σ	m	ρ	σ
0	0.99819	0.00001	0	0.99819	0.00001	0	0.99819	0.00001
$9.971\cdot10^{-7}$	0.99819	0.00003	$1.220\cdot10^{-6}$	0.99819	0.00001	$1.010\cdot10^{-6}$	0.99820	0.00001
$1.400\cdot10^{-5}$	0.99820	0.00003	$1.550\cdot10^{-4}$	0.99821	0.00001	$9.990\cdot10^{-6}$	0.99820	0.00001
$1.790\cdot10^{-4}$	0.99820	0.00001	$9.980\cdot10^{-4}$	0.99831	0.00001	$1.010\cdot10^{-4}$	0.99821	0.00001
$1.039\cdot10^{-3}$	0.99826	0.00001	$9.993\cdot10^{-3}$	0.99925	0.00001	$1.127\cdot10^{-3}$	0.99837	0.00001
$9.271\cdot10^{-3}$	0.99890	0.00003	$2.010\cdot10^{-2}$	1.00060	0.00031	$9.993\cdot10^{-3}$	1.00002	0.00001
$1.980\cdot10^{-2}$	0.99964	0.00001	$4.090\cdot10^{-2}$	1.00280	0.00001	$1.980\cdot10^{-2}$	1.00150	0.00001

$3.820\cdot10^{-2}$	1.00010	0.00001	$6.030\cdot10^{-2}$	1.00530	0.00001	$3.830\cdot10^{-2}$	1.00410	0.00014
$6.070\cdot10^{-2}$	1.00250	0.00003	$8.170\cdot10^{-2}$	1.00770	0.00018	$5.870\cdot10^{-2}$	1.00840	0.00001
$7.930\cdot10^{-2}$	1.00380	0.00019	$9.992\cdot10^{-2}$	1.00988	0.00001	$7.810\cdot10^{-2}$	1.01040	0.00018
$9.986\cdot10^{-2}$	1.00561	0.00001	$1.210\cdot10^{-1}$	1.01230	0.00001	$9.993\cdot10^{-2}$	1.01544	0.00001
$1.180\cdot10^{-1}$	1.00650	0.00001	$1.370\cdot10^{-1}$	1.01410	0.00031	$1.160\cdot10^{-1}$	1.01910	0.00004
$1.350\cdot10^{-1}$	1.00770	0.00001	$1.650\cdot10^{-1}$	1.01740	0.00001	$1.400\cdot10^{-1}$	1.02010	0.00018
$1.640\cdot10^{-1}$	1.00970	0.00001	$1.984\cdot10^{-1}$	1.02146	0.00001	$1.630\cdot10^{-1}$	1.02630	0.00003
$1.750\cdot10^{-1}$	1.01050	0.00017	$2.510\cdot10^{-1}$	1.02750	0.00015	$1.960\cdot10^{-1}$	1.03020	0.00016
$1.985\cdot10^{-1}$	1.01223	0.00001	$2.940\cdot10^{-1}$	1.03130	0.00001	$1.986\cdot10^{-1}$	1.03167	0.00001
$2.590\cdot10^{-1}$	1.01580	0.00001	$2.963\cdot10^{-1}$	1.03281	0.00003	$2.470\cdot10^{-1}$	1.04040	0.00001
$2.910\cdot10^{-1}$	1.01830	0.00001	$3.900\cdot10^{-1}$	1.04330	0.00001	$2.523\cdot10^{-1}$	1.04045	0.00001
$2.984\cdot10^{-1}$	1.01892	0.00003	$3.994\cdot10^{-1}$	1.04404	0.00003	$2.960\cdot10^{-1}$	1.04740	0.00020
$3.990\cdot10^{-1}$	1.02590	0.00001	$5.037\cdot10^{-1}$	1.05597	0.00001	$2.988\cdot10^{-1}$	1.04912	0.00001
$3.995\cdot10^{-1}$	1.02592	0.00003	$7.983\cdot10^{-1}$	1.08848	0.00001	$3.973\cdot10^{-1}$	1.06525	0.00001
$5.015\cdot10^{-1}$	1.03270	0.00003	1.001	1.10938	0.00001	$4.524\cdot10^{-1}$	1.07438	0.00001
$7.427\cdot10^{-1}$	1.04864	0.00003	2.001	1.20863	0.00001			
1.000	1.06493	0.00001	2.296	1.23742	0.00001			
1.997	1.12534	0.00001						
3.980	1.22321	0.00006						
5.989	1.31273	0.00003						
7.996	1.38852	0.00001						
9.022	1.42687	0.00003						

Table S2. Density (ρ, in g·cm^{-3}) at 25° C of sodium chlorate, sodium bromate and sodium iodate solutions at different concentrations (*m*, in molal units). σ is the standard deviation.

$NaClO_3$			$NaBrO_3$			$NaIO_3$		
m	ρ	σ	*m*	ρ	σ	*m*	ρ	σ
0	0.99704	0.00001	0	0.99704	0.00001	0	0.99704	0.00001
$9.971\cdot10^{-7}$	0.99704	0.00001	$1.220\cdot10^{-6}$	0.99704	0.00001	$1.010\cdot10^{-6}$	0.99704	0.00001
$1.400\cdot10^{-5}$	0.99704	0.00001	$1.400\cdot10^{-5}$	0.99704	0.00001	$9.990\cdot10^{-6}$	0.99704	0.00001
$1.790\cdot10^{-4}$	0.99704	0.00001	$1.550\cdot10^{-4}$	0.99705	0.00001	$1.010\cdot10^{-4}$	0.99706	0.00001
$1.039\cdot10^{-3}$	0.99709	0.00001	$9.980\cdot10^{-4}$	0.99715	0.00001	$1.127\cdot10^{-3}$	0.99721	0.00001
$9.271\cdot10^{-3}$	0.99773	0.00001	$9.993\cdot10^{-3}$	0.99809	0.00001	$9.993\cdot10^{-3}$	0.99884	0.00001
$1.980\cdot10^{-2}$	0.99847	0.00001	$2.010\cdot10^{-2}$	0.99944	0.00031	$1.980\cdot10^{-2}$	1.00030	0.00001
$3.820\cdot10^{-2}$	0.99980	0.00001	$4.090\cdot10^{-2}$	1.00160	0.00001	$4.040\cdot10^{-2}$	1.00310	0.00001
$6.070\cdot10^{-2}$	1.00130	0.00001	$6.030\cdot10^{-2}$	1.00410	0.00001	$5.870\cdot10^{-2}$	1.00830	0.00012
$7.930\cdot10^{-2}$	1.00260	0.00001	$8.170\cdot10^{-2}$	1.00650	0.00018	$8.080\cdot10^{-2}$	1.01080	0.00001
$9.986\cdot10^{-2}$	1.00435	0.00001	$9.992\cdot10^{-2}$	1.00862	0.00001	$9.993\cdot10^{-2}$	1.01414	0.00001
$1.180\cdot10^{-1}$	1.00520	0.00001	$1.210\cdot10^{-1}$	1.01100	0.00001	$1.160\cdot10^{-1}$	1.01610	0.00001
$1.350\cdot10^{-1}$	1.00650	0.00001	$1.370\cdot10^{-1}$	1.01280	0.00031	$1.450\cdot10^{-1}$	1.02150	0.00001
$1.640\cdot10^{-1}$	1.00840	0.00001	$1.650\cdot10^{-1}$	1.01610	0.00001	$1.630\cdot10^{-1}$	1.02490	0.00001
$1.750\cdot10^{-1}$	1.00920	0.00001	$1.984\cdot10^{-1}$	1.02011	0.00001	$1.960\cdot10^{-1}$	1.02966	0.00020
$1.985\cdot10^{-1}$	1.01090	0.00001	$2.510\cdot10^{-1}$	1.02600	0.00002	$1.986\cdot10^{-1}$	1.03025	0.00001
$2.590\cdot10^{-1}$	1.01490	0.00001	$2.940\cdot10^{-1}$	1.02910	0.00015	$2.470\cdot10^{-1}$	1.03890	0.00001
$2.910\cdot10^{-1}$	1.01690	0.00001	$2.963\cdot10^{-1}$	1.03136	0.00001	$2.523\cdot10^{-1}$	1.03893	0.00001
$2.984\cdot10^{-1}$	1.01750	0.00001	$3.900\cdot10^{-1}$	1.04180	0.00001	$2.988\cdot10^{-1}$	1.04758	0.00001
$3.990\cdot10^{-1}$	1.02440	0.00001	$3.994\cdot10^{-1}$	1.04250	0.00001	$3.030\cdot10^{-1}$	1.04810	0.00001
$3.995\cdot10^{-1}$	1.02442	0.00001	$5.037\cdot10^{-1}$	1.05435	0.00001	$3.990\cdot10^{-1}$	1.06430	0.00001
$5.015\cdot10^{-1}$	1.03112	0.00001	$7.983\cdot10^{-1}$	1.08661	0.00001	$4.524\cdot10^{-1}$	1.07266	0.00001
$7.427\cdot10^{-1}$	1.04689	0.00001	1.001	1.10736	0.00001			
1.000	1.06301	0.00001	2.001	1.20596	0.00001			
1.997	1.12286	0.00001	2.296	1.23459	0.00001			
3.980	1.21940	0.00001						
5.989	1.30914	0.00001						
7.996	1.38458	0.00001						

NaClO$_3$			NaBrO$_3$			NaIO$_3$		
m	*ρ*	*σ*	*m*	*ρ*	*σ*	*m*	*ρ*	*σ*
9.022	1.42285	0.00001						

Table S3. Density (ρ, in g·cm^{-3}) at 30° C of sodium chlorate, sodium bromate and sodium iodate solutions at different concentrations (m, in molal units). σ is the standard deviation.

NaClO$_3$			NaBrO$_3$			NaIO$_3$		
m	*ρ*	*σ*	*m*	*ρ*	*σ*	*m*	*ρ*	*σ*
0	0.99564	0.00001	0	0.99564	0.00001	0	0.99564	0.00001
$1.790 \cdot 10^{-4}$	0.99565	0.00004	$1.220 \cdot 10^{-6}$	0.99564	0.00001	$1.010 \cdot 10^{-6}$	0.99564	0.00001
$9.271 \cdot 10^{-3}$	0.99628	0.00003	$1.400 \cdot 10^{-5}$	0.99564	0.00001	$9.990 \cdot 10^{-6}$	0.99565	0.00001
$1.980 \cdot 10^{-2}$	0.99706	0.00001	$1.550 \cdot 10^{-4}$	0.99566	0.00001	$1.010 \cdot 10^{-3}$	0.99566	0.00001
$3.820 \cdot 10^{-2}$	0.99838	0.00001	$9.980 \cdot 10^{-4}$	0.99575	0.00001	$1.127 \cdot 10^{-3}$	0.99582	0.00001
$6.070 \cdot 10^{-2}$	0.99983	0.00001	$9.993 \cdot 10^{-3}$	0.99668	0.00014	$9.993 \cdot 10^{-3}$	0.99744	0.00001
$7.930 \cdot 10^{-2}$	1.00110	0.00001	$2.010 \cdot 10^{-2}$	0.99803	0.00010	$1.980 \cdot 10^{-2}$	0.99889	0.00001
$9.986 \cdot 10^{-2}$	1.00279	0.00005	$4.090 \cdot 10^{-2}$	1.00020	0.00001	$4.040 \cdot 10^{-2}$	1.00250	0.00001
$1.180 \cdot 10^{-1}$	1.00370	0.00001	$6.030 \cdot 10^{-2}$	1.00260	0.00001	$5.870 \cdot 10^{-2}$	1.00570	0.00001
$1.350 \cdot 10^{-1}$	1.00410	0.00001	$8.170 \cdot 10^{-2}$	1.00500	0.00019	$8.080 \cdot 10^{-2}$	1.00930	0.00001
$1.640 \cdot 10^{-1}$	1.00690	0.00001	$9.992 \cdot 10^{-2}$	1.00714	0.00001	$9.993 \cdot 10^{-2}$	1.01262	0.00001
$1.750 \cdot 10^{-1}$	1.00760	0.00001	$1.210 \cdot 10^{-1}$	1.00950	0.00001	$1.160 \cdot 10^{-1}$	1.01540	0.00001
$1.985 \cdot 10^{-1}$	1.00926	0.00006	$1.370 \cdot 10^{-1}$	1.01130	0.00010	$1.450 \cdot 10^{-1}$	1.01990	0.00001
$2.590 \cdot 10^{-1}$	1.01330	0.00001	$1.620 \cdot 10^{-1}$	1.01410	0.00001	$1.630 \cdot 10^{-1}$	1.02320	0.00003
$2.910 \cdot 10^{-1}$	1.01530	0.00001	$1.984 \cdot 10^{-1}$	1.01853	0.00001	$1.986 \cdot 10^{-1}$	1.02862	0.00001
$2.984 \cdot 10^{-1}$	1.01584	0.00003	$2.510 \cdot 10^{-1}$	1.02440	0.00002	$2.470 \cdot 10^{-1}$	1.03720	0.00001
$3.990 \cdot 10^{-1}$	1.02270	0.00001	$2.963 \cdot 10^{-1}$	1.02970	0.00001	$2.523 \cdot 10^{-1}$	1.03765	0.00001
$5.015 \cdot 10^{-1}$	1.02925	0.00007	$3.900 \cdot 10^{-1}$	1.04000	0.00001	$2.988 \cdot 10^{-1}$	1.04582	0.00001
$7.427 \cdot 10^{-1}$	1.04480	0.00034	$3.994 \cdot 10^{-1}$	1.04076	0.00001	$3.030 \cdot 10^{-1}$	1.04640	0.00001
1.000	1.06093	0.00001	$5.037 \cdot 10^{-1}$	1.05252	0.00001	$3.973 \cdot 10^{-1}$	1.06173	0.00001
1.997	1.12025	0.00001	$7.983 \cdot 10^{-1}$	1.08456	0.00001	$3.990 \cdot 10^{-1}$	1.06240	0.00001
3.980	1.21618	0.00001	1.001	1.10517	0.00001	$4.524 \cdot 10^{-1}$	1.07074	0.00001
5.989	1.30548	0.00001	2.001	1.20318	0.00001			
7.996	1.38057	0.00001	2.296	1.23165	0.00001			
9.022	1.41879	0.00001						

Table S4. Density (ρ, in g·cm^{-3}) at 35° C of sodium chlorate, sodium bromate and sodium iodate solutions at different concentrations (m, in molal units). σ is the standard deviation.

**$NaClO_3$**			**$NaBrO_3$**			**$NaIO_3$**		
m	ρ	σ	m	ρ	σ	m	ρ	σ
0	0.99403	0.00001	0	0.99403	0.00001	0	0.99403	0.00001
$1.790 \cdot 10^{-4}$	0.99403	0.00000	$1.550 \cdot 10^{-4}$	0.99403	0.00003	$1.010 \cdot 10^{-4}$	0.99405	0.00002
$9.271 \cdot 10^{-3}$	0.99433	0.00011	$9.993 \cdot 10^{-3}$	0.99487	0.00014	$1.127 \cdot 10^{-3}$	0.99410	0.00009
$1.980 \cdot 10^{-2}$	0.99542	0.00001	$2.010 \cdot 10^{-2}$	0.99639	0.00001	$9.993 \cdot 10^{-3}$	0.99563	0.00017
$3.820 \cdot 10^{-2}$	0.99674	0.00001	$4.090 \cdot 10^{-2}$	0.99845	0.00008	$1.980 \cdot 10^{-2}$	0.99723	0.00116
$6.070 \cdot 10^{-2}$	0.99814	0.00002	$6.030 \cdot 10^{-2}$	1.00070	0.00012	$4.040 \cdot 10^{-2}$	1.00090	0.00001
$7.930 \cdot 10^{-2}$	0.99942	0.00029	$8.170 \cdot 10^{-2}$	1.00330	0.00035	$5.870 \cdot 10^{-2}$	1.00380	0.00017
$9.986 \cdot 10^{-2}$	1.00073	0.00012	$9.992 \cdot 10^{-2}$	1.00533	0.00026	$8.080 \cdot 10^{-2}$	1.00760	0.00001
$1.180 \cdot 10^{-1}$	1.00200	0.00001	$1.210 \cdot 10^{-1}$	1.00660	0.00014	$9.993 \cdot 10^{-2}$	1.01090	0.00009
$1.350 \cdot 10^{-1}$	1.00300	0.00017	$1.390 \cdot 10^{-1}$	1.00970	0.00001	$1.160 \cdot 10^{-1}$	1.01370	0.00004
$1.640 \cdot 10^{-1}$	1.00520	0.00001	$1.650 \cdot 10^{-1}$	1.01170	0.00041	$1.450 \cdot 10^{-1}$	1.01810	0.00001
$1.985 \cdot 10^{-1}$	1.00712	0.00013	$1.984 \cdot 10^{-1}$	1.01646	0.00010	$1.630 \cdot 10^{-1}$	1.02070	0.00045
$2.590 \cdot 10^{-1}$	1.01150	0.00001	$2.510 \cdot 10^{-1}$	1.02240	0.00003	$1.986 \cdot 10^{-1}$	1.02675	0.00009
$2.910 \cdot 10^{-1}$	1.01350	0.00001	$2.940 \cdot 10^{-1}$	1.02600	0.00041	$2.470 \cdot 10^{-1}$	1.03490	0.00031
$2.984 \cdot 10^{-1}$	1.01371	0.00010	$2.963 \cdot 10^{-1}$	1.02752	0.00034	$2.523 \cdot 10^{-1}$	1.03530	0.00001
$3.990 \cdot 10^{-1}$	1.02080	0.00001	$3.900 \cdot 10^{-1}$	1.03810	0.00001	$2.988 \cdot 10^{-1}$	1.04387	0.00001
$5.015 \cdot 10^{-1}$	1.02658	0.00028	$3.994 \cdot 10^{-1}$	1.03884	0.00001	$3.030 \cdot 10^{-1}$	1.04440	0.00001
$7.427 \cdot 10^{-1}$	1.04248	0.00014	$5.037 \cdot 10^{-1}$	1.05052	0.00001	$3.973 \cdot 10^{-1}$	1.05968	0.00001
1.000	1.05867	0.00001	$7.983 \cdot 10^{-1}$	1.08236	0.00001	$3.990 \cdot 10^{-1}$	1.06040	0.00001
1.997	1.11726	0.00014	1.001	1.10285	0.00001	$4.524 \cdot 10^{-1}$	1.06864	0.00001
3.980	1.21289	0.00001	2.001	1.20030	0.00001			
5.989	1.30177	0.00001	2.296	1.22861	0.00001			
7.996	1.37648	0.00002						
9.022	1.41471	0.00001						

Table S5. Density (ρ, in g·cm^{-3}) at 40° C of sodium chlorate, sodium bromate and sodium iodate solutions at different concentrations (m, in molal units). σ is the standard deviation.

$NaClO_3$			$NaBrO_3$			$NaIO_3$		
m	ρ	σ	m	ρ	σ	m	ρ	σ
0	0.99222	0.00001	0	0.99222	0.00001	0	0.99222	0.00001
$1.400 \cdot 10^{-5}$	0.99222	0.00008	$1.550 \cdot 10^{-4}$	0.99223	0.00022	$1.010 \cdot 10^{-4}$	0.99222	0.00051
$1.790 \cdot 10^{-5}$	0.99222	0.00008	$9.993 \cdot 10^{-3}$	0.99224	0.00062	$9.993 \cdot 10^{-3}$	0.99266	0.00002
$9.271 \cdot 10^{-3}$	0.99224	0.00013	$2.010 \cdot 10^{-2}$	0.99466	0.00030	$2.120 \cdot 10^{-2}$	0.99576	0.00001
$1.980 \cdot 10^{-2}$	0.99227	0.00001	$4.090 \cdot 10^{-2}$	0.99666	0.00043	$4.170 \cdot 10^{-2}$	0.99907	0.00001
$3.820 \cdot 10^{-2}$	0.99335	0.00016	$6.030 \cdot 10^{-2}$	0.99880	0.00057	$6.020 \cdot 10^{-2}$	1.00210	0.00001
$6.070 \cdot 10^{-2}$	0.99480	0.00014	$8.170 \cdot 10^{-2}$	1.00120	0.00014	$8.080 \cdot 10^{-2}$	1.00490	0.00005
$7.930 \cdot 10^{-2}$	0.99592	0.00020	$9.992 \cdot 10^{-2}$	1.00235	0.00057	$9.993 \cdot 10^{-2}$	1.00897	0.00002
$9.986 \cdot 10^{-2}$	0.99840	0.00010	$1.210 \cdot 10^{-1}$	1.00500	0.00051	$1.160 \cdot 10^{-1}$	1.01170	0.00001
$1.390 \cdot 10^{-1}$	1.00010	0.00001	$1.390 \cdot 10^{-1}$	1.00780	0.00001	$1.450 \cdot 10^{-1}$	1.01640	0.00013
$1.620 \cdot 10^{-1}$	1.00160	0.00001	$1.650 \cdot 10^{-1}$	1.00910	0.00034	$1.590 \cdot 10^{-1}$	1.01860	0.00001
$1.750 \cdot 10^{-1}$	1.00280	0.00016	$1.984 \cdot 10^{-1}$	1.01271	0.00063	$1.960 \cdot 10^{-1}$	1.02410	0.00057
$1.985 \cdot 10^{-1}$	1.00445	0.00021	$2.320 \cdot 10^{-1}$	1.01790	0.00001	$1.986 \cdot 10^{-1}$	1.02453	0.00002
$2.890 \cdot 10^{-1}$	1.00950	0.00001	$2.510 \cdot 10^{-1}$	1.01990	0.00037	$2.470 \cdot 10^{-1}$	1.03190	0.00043
$2.984 \cdot 10^{-1}$	1.01127	0.00015	$2.940 \cdot 10^{-1}$	1.02510	0.00005	$2.523 \cdot 10^{-1}$	1.03285	0.00004
$3.990 \cdot 10^{-1}$	1.01150	0.00001	$3.900 \cdot 10^{-1}$	1.03600	0.00001	$2.988 \cdot 10^{-1}$	1.04165	0.00007
$3.995 \cdot 10^{-1}$	1.01781	0.00019	$3.994 \cdot 10^{-1}$	1.03641	0.00031	$3.973 \cdot 10^{-1}$	1.05738	0.00007
$5.015 \cdot 10^{-1}$	1.02353	0.00028	$5.037 \cdot 10^{-1}$	1.04821	0.00005	$3.990 \cdot 10^{-1}$	1.05790	0.00013
$7.427 \cdot 10^{-1}$	1.03964	0.00017	$7.983 \cdot 10^{-1}$	1.07998	0.00001	$4.524 \cdot 10^{-1}$	1.06626	0.00007
1.000	1.05614	0.00007	1.001	1.10036	0.00001			
1.997	1.11359	0.00026	2.001	1.19731	0.00001			
3.980	1.20954	0.00001	2.296	1.22543	0.00002			
5.989	1.29803	0.00001						
7.996	1.37234	0.00003						
9.022	1.41058	0.00001						

Table S6. Standard partial molar volumes ($\overline{V}_2^{o}$, in cm^3·mol^{-1}) at 20°, 25°, 30°, 35°, 40° C for sodium chlorate, bromate and iodate.

T	$NaClO_3$	$NaBrO_3$	$NaIO_3$
20° C	34.9 ± 0.2	31.9 ± 0.3	23.4 ± 0.3
25° C	35.7 ± 0.2	32.8 ± 0.2	24.7 ± 0.3
30° C	36.5 ± 0.1	33.8 ± 0.2	25.7 ± 0.3
35° C	38.3 ± 0.3	35.0 ± 0.3	26.7 ± 0.3
40° C	40.5 ± 0.2	35.8 ± 0.5	27.6 ± 0.2

Table S7. Viscosity (η, in cP) at 25° C of sodium chlorate, bromate and iodate solutions at different concentrations (*m*, in molal units). σ indicates the standard deviation.

$NaClO_3$			$NaBrO_3$			$NaIO_3$		
m	η	σ	*m*	η	σ	*m*	η	σ
0	0.890	0.001	0	0.890	0.001	0	0.890	0.001
$1.016\cdot10^{-2}$	0.890	0.001	$1.004\cdot10^{-2}$	0.890	0.002	$9.986\cdot10^{-3}$	0.892	0.001
$1.113\cdot10^{-1}$	0.900	0.002	$1.003\cdot10^{-1}$	0.898	0.001	$1.006\cdot10^{-1}$	0.911	0.002
$2.031\cdot10^{-1}$	0.903	0.002	$2.033\cdot10^{-1}$	0.907	0.001	$1.986\cdot10^{-1}$	0.931	0.001
$3.089\cdot10^{-1}$	0.910	0.001	$2.993\cdot10^{-1}$	0.916	0.001	$2.502\cdot10^{-1}$	0.944	0.001
$4.001\cdot10^{-1}$	0.916	0.002	$4.018\cdot10^{-1}$	0.929	0.002	$2.988\cdot10^{-1}$	0.954	0.001
$5.003\cdot10^{-1}$	0.927	0.002	$5.037\cdot10^{-1}$	0.939	0.001	$3.973\cdot10^{-1}$	0.977	0.001
$7.337\cdot10^{-1}$	0.940	0.002	$7.983\cdot10^{-1}$	0.968	0.001	$4.524\cdot10^{-1}$	0.990	0.004
$9.968\cdot10^{-1}$	0.964	0.001	1.001	1.001	0.003			
1.983	1.062	0.002	2.001	1.139	0.001			
3.980	1.344	0.003	2.296	1.186	0.001			
5.989	1.702	0.003						
7.996	2.210	0.003						
9.022	2.481	0.001						

Table S8. Conductivity (κ, in $\mu S\cdot cm^{-1}$) at 25° C of sodium chlorate, bromate and iodate solutions at different concentrations (*c*, in molar units). σ is the standard deviation.

$NaClO_3$			$NaBrO_3$			$NaIO_3$		
c	κ	σ	*c*	κ	σ	*c*	κ	σ
0	0.84	0.10	0	0.84	0.10	0	0.84	0.10
$9.941\cdot10^{-7}$	0.98	0.10	$1.216\cdot10^{-6}$	0.98	0.01	$1.010\cdot10^{-6}$	1.10	0.01
$1.396\cdot10^{-5}$	2.46	0.01	$1.396\cdot10^{-5}$	2.15	0.01	$9.990\cdot10^{-6}$	2.92	0.01
$1.785\cdot10^{-4}$	13.55	0.01	$1.545\cdot10^{-4}$	15.51	0.01	$1.010\cdot10^{-4}$	10.74	0.01
$1.036\cdot10^{-3}$	118.70	0.10	$9.950\cdot10^{-4}$	109.20	0.10	$1.127\cdot10^{-3}$	93.40	0.10

$9.241\cdot10^{-3}$	1119.00	1.00	$9.959\cdot10^{-3}$	1047.00	1.00	$9.993\cdot10^{-3}$	881.00	1.00
$9.924\cdot10^{-2}$	9680.00	10.00	$9.928\cdot10^{-2}$	8860.00	10.00	$9.993\cdot10^{-2}$	7240.00	10.00
$1.965\cdot10^{-1}$	17710.00	10.00	$1.965\cdot10^{-1}$	16270.00	10.00	$1.986\cdot10^{-1}$	12760.00	10.00
$2.943\cdot10^{-1}$	25900.00	10.00	$2.925\cdot10^{-1}$	23200.00	10.00	$2.523\cdot10^{-1}$	15263.33	11.55
$3.925\cdot10^{-1}$	33500.00	10.00	$3.927\cdot10^{-1}$	30200.00	10.00	$2.988\cdot10^{-1}$	17846.67	57.74
$4.909\cdot10^{-1}$	37866.67	57.74	$4.936\cdot10^{-1}$	34800.00	50.12	$3.973\cdot10^{-1}$	22700.00	57.74
$7.205\cdot10^{-1}$	49515.99	57.74	$7.742\cdot10^{-1}$	50900.00	50.12	$4.524\cdot10^{-1}$	24966.67	57.74
$9.610\cdot10^{-1}$	65466.67	57.74	$9.633\cdot10^{-1}$	60400.00	50.12			
1.849	104200.00	57.74	1.853	95600.00	50.12			
3.409	151266.67	57.74	2.106	102833.33	57.74			
4.788	161266.67	57.74						
5.980	156933.33	57.74						
6.549	151466.67	57.74						

Table S9. Molar conductivity (Λ, in S·cm^2·mol^{-1}) at 25° C for sodium chlorate, sodium bromate and sodium iodate solutions at different concentrations (*c*, in molar units). σ is the standard deviation.

**$NaClO_3$**			**$NaBrO_3$**			**$NaIO_3$**		
c	Λ	σ	*c*	Λ	σ	*c*	Λ	σ
$1.036\cdot10^{-3}$	114.59	0.10	$9.950\cdot10^{-4}$	109.75	0.10	$1.600\cdot10^{-3}$	92.00	0.01
$9.924\cdot10^{-2}$	97.54	0.10	$9.959\cdot10^{-3}$	105.13	0.10	$9.993\cdot10^{-3}$	88.16	0.10
$1.965\cdot10^{-1}$	90.13	0.05	$9.928\cdot10^{-2}$	89.24	0.10	$5.025\cdot10^{-2}$	80.55	0.02
$2.943\cdot10^{-1}$	88.01	0.03	$1.965\cdot10^{-1}$	82.81	0.05	$9.993\cdot10^{-2}$	72.45	0.10
$3.925\cdot10^{-1}$	85.35	0.03	$4.936\cdot10^{-1}$	70.51	0.10	$1.407\cdot10^{-1}$	68.03	0.08
1.849	56.35	0.03	$7.742\cdot10^{-1}$	65.74	0.06	$1.986\cdot10^{-1}$	64.25	0.05
3.409	44.37	0.02	$9.633\cdot10^{-1}$	62.70	0.05	$2.523\cdot10^{-1}$	60.49	0.05
4.788	33.68	0.01	1.853	51.58	0.03	$2.988\cdot10^{-1}$	59.74	0.02
5.981	26.24	0.01	2.106	48.84	0.03	$3.518\cdot10^{-1}$	58.06	0.02
6.549	23.13	0.01				$3.973\cdot10^{-1}$	57.14	0.01
						$4.524\cdot10^{-1}$	55.19	0.13

Table S10. Mean ionic activity coefficients ($\gamma_{\pm}$) at 25° C of sodium chlorate, sodium bromate and sodium iodate solutions as a function of the concentration (c, in molar units) obtained by using eq. A9. The error is of 1.3%, 0.6% and 2.1% for sodium chlorate, bromate and iodate, respectively.

**$NaClO_3$**				**$NaBrO_3$**				**$NaIO_3$**			
c	$\gamma_{\pm}$	c^a	$\gamma_{\pm}^a$	c	$\gamma_{\pm}$	c^a	$\gamma_{\pm}^a$	c	$\gamma_{\pm}$	c^b	$\gamma_{\pm}^b$
$1.036\cdot10^{-3}$	1.064	$9.970\cdot10^{-4}$	0.965	$9.950\cdot10^{-4}$	0.967	$9.970\cdot10^{-4}$	0.965	$1.127\cdot10^{-3}$	0.946	$1.992\cdot10^{-2}$	0.854
$9.241\cdot10^{-3}$	0.974	$1.994\cdot10^{-3}$	0.952	$9.959\cdot10^{-3}$	0.908	$1.994\cdot10^{-3}$	0.951	$9.993\cdot10^{-3}$	0.877	$4.974\cdot10^{-2}$	0.787
$9.924\cdot10^{-2}$	0.775	$4.984\cdot10^{-3}$	0.927	$9.928\cdot10^{-2}$	0.731	$4.984\cdot10^{-3}$	0.926	$9.993\cdot10^{-2}$	0.755	$9.925\cdot10^{-2}$	0.725
$1.965\cdot10^{-1}$	0.701	$9.966\cdot10^{-3}$	0.902	$1.965\cdot10^{-1}$	0.670	$9.966\cdot10^{-3}$	0.900	$1.986\cdot10^{-1}$	0.671	$1.976\cdot10^{-1}$	0.650
$2.943\cdot10^{-1}$	0.665	$1.992\cdot10^{-2}$	0.870	$2.925\cdot10^{-1}$	0.639	$1.992\cdot10^{-2}$	0.867	$2.523\cdot10^{-1}$	0.635	$2.950\cdot10^{-1}$	0.597
$3.925\cdot10^{-1}$	0.632	$4.975\cdot10^{-2}$	0.818	$3.927\cdot10^{-1}$	0.610	$4.974\cdot10^{-2}$	0.811	$2.988\cdot10^{-1}$	0.623	$3.916\cdot10^{-1}$	0.557
$4.909\cdot10^{-1}$	0.588	$9.928\cdot10^{-2}$	0.772	$4.936\cdot10^{-1}$	0.578	$9.926\cdot10^{-2}$	0.758	$3.973\cdot10^{-1}$	0.605		
$7.205\cdot10^{-1}$	0.544	$1.977\cdot10^{-1}$	0.720	$7.742\cdot10^{-1}$	0.531	$1.976\cdot10^{-1}$	0.696	$4.524\cdot10^{-1}$	0.589		
$9.610\cdot10^{-1}$	0.521	$2.954\cdot10^{-1}$	0.688	$9.633\cdot10^{-1}$	0.509	$2.951\cdot10^{-1}$	0.657				
1.849	0.445	$3.922\cdot10^{-1}$	0.664	1.853	0.438	$3.918\cdot10^{-1}$	0.628				
3.409	0.378	$4.882\cdot10^{-1}$	0.645	2.106	0.422	$4.875\cdot10^{-1}$	0.605				
4.788	0.328	$5.834\cdot10^{-1}$	0.630			$5.825\cdot10^{-1}$	0.585				
5.981	0.296	$6.778\cdot10^{-1}$	0.617			$6.766\cdot10^{-1}$	0.569				
6.549	0.284	$7.714\cdot10^{-1}$	0.606			$7.699\cdot10^{-1}$	0.554				
		$8.643\cdot10^{-1}$	0.597			$8.624\cdot10^{-1}$	0.541				
		$9.564\cdot10^{-1}$	0.589			$9.541\cdot10^{-1}$	0.528				
		1.138	0.575								
		1.318	0.563								
		1.494	0.553								
		1.667	0.545								
		1.838	0.538								
		2.253	0.525								
		2.653	0.515								
		3.038	0.508								

a: Data from Refs. [49] and [88], b: Data from Ref. [89].

Table S11. Refractive index (*n*) at 20° C of sodium chlorate, bromate and iodate solutions at different concentrations (*c*, in molar units and *c** , in g·mL^{-1}). σ is the standard deviation.

$NaClO_3$				$NaBrO_3$				$NaIO_3$			
c	*c**	*n*	σ	*c*	*c**	*n*	σ	*c*	*c**	*n*	σ
0	0	1.332	0.001	0	0	1.332	0.001	0	0	1.332	0.001
$4.729 \cdot 10^{-1}$	$5.033 \cdot 10^{-2}$	1.337	0.001	$1.052 \cdot 10^{-1}$	$1.587 \cdot 10^{-2}$	1.334	0.001	$9.401 \cdot 10^{-2}$	$1.860 \cdot 10^{-2}$	1.334	0.001
$8.717 \cdot 10^{-1}$	$9.279 \cdot 10^{-2}$	1.340	0.001	$1.898 \cdot 10^{-1}$	$2.864 \cdot 10^{-2}$	1.335	0.001	$1.927 \cdot 10^{-1}$	$3.813 \cdot 10^{-2}$	1.336	0.001
1.787	$1.902 \cdot 10^{-1}$	1.349	0.001	$2.718 \cdot 10^{-1}$	$4.101 \cdot 10^{-2}$	1.336	0.001	$2.571 \cdot 10^{-1}$	$5.088 \cdot 10^{-2}$	1.338	0.001
2.535	$2.698 \cdot 10^{-1}$	1.355	0.001	$3.850 \cdot 10^{-1}$	$5.810 \cdot 10^{-2}$	1.338	0.001	$3.695 \cdot 10^{-1}$	$7.311 \cdot 10^{-2}$	1.341	0.001
3.395	$3.613 \cdot 10^{-1}$	1.361	0.001	$4.837 \cdot 10^{-1}$	$7.299 \cdot 10^{-2}$	1.340	0.001	$4.673 \cdot 10^{-1}$	$9.247 \cdot 10^{-2}$	1.343	0.001
3.948	$4.202 \cdot 10^{-1}$	1.367	0.001	$5.475 \cdot 10^{-1}$	$8.261 \cdot 10^{-2}$	1.341	0.001				
4.685	$4.987 \cdot 10^{-1}$	1.372	0.001	$6.728 \cdot 10^{-1}$	$1.015 \cdot 10^{-1}$	1.343	0.001				
5.463	$5.815 \cdot 10^{-1}$	1.379	0.001	$7.411 \cdot 10^{-1}$	$1.118 \cdot 10^{-1}$	1.344	0.001				
5.938	$6.320 \cdot 10^{-1}$	1.383	0.001								
